# Immunotherapeutic Concepts to Target Acute Myeloid Leukemia: Focusing on the Role of Monoclonal Antibodies, Hypomethylating Agents and the Leukemic Microenvironment

**DOI:** 10.3390/ijms18081660

**Published:** 2017-07-31

**Authors:** Olumide Babajide Gbolahan, Amer M. Zeidan, Maximilian Stahl, Mohammad Abu Zaid, Sherif Farag, Sophie Paczesny, Heiko Konig

**Affiliations:** 1 Department of Medicine, Division of Hematology/Oncology, Indiana University School of Medicine, Indianapolis, IN 46202, USA; obgbolah@iu.edu; 2 Department of Medicine, Section of Hematology, Yale University School of Medicine, New Haven, CT 06510, USA; amer.zeidan@yale.edu (A.M.Z.); maximilian.stahl@yale.edu (M.S.); 3 Department of Medicine, Bone Marrow and Stem Cell Transplantation, Indiana University School of Medicine, Indianapolis, IN 46202, USA; mabuzaid@iu.edu (M.A.Z.); ssfarag@iu.edu (S.F.); 4 Wells Center for Pediatric Research, Riley Hospital for Children, Indiana University School of Medicine, Indianapolis, IN 46202, USA; sophpacz@iu.edu

**Keywords:** acute myeloid leukemia, immunotherapy, monoclonal antibodies, hypomethylating agents, microenvironment

## Abstract

Intensive chemotherapeutic protocols and allogeneic stem cell transplantation continue to represent the mainstay of acute myeloid leukemia (AML) treatment. Although this approach leads to remissions in the majority of patients, long-term disease control remains unsatisfactory as mirrored by overall survival rates of approximately 30%. The reason for this poor outcome is, in part, due to various toxicities associated with traditional AML therapy and the limited ability of most patients to tolerate such treatment. More effective and less toxic therapies therefore represent an unmet need in the management of AML, a disease for which therapeutic progress has been traditionally slow when compared to other cancers. Several studies have shown that leukemic blasts elicit immune responses that could be exploited for the development of novel treatment concepts. To this end, early phase studies of immune-based therapies in AML have delivered encouraging results and demonstrated safety and feasibility. In this review, we discuss opportunities for immunotherapeutic interventions to enhance the potential to achieve a cure in AML, thereby focusing on the role of monoclonal antibodies, hypomethylating agents and the leukemic microenvironment.

## 1. Introduction

Acute myeloid leukemia (AML) remains one of the greatest therapeutic challenges in the field of hematologic malignancies. Despite significant progress in understanding AML at the molecular level, current AML treatments almost generally fail following an initial remission and have remained largely unchanged for almost 40 years [1]. Only about 35–40% of adult patients aged 60 years or younger and approximately 5–15% of elderly patients are currently cured by the means of conventional anti-leukemic treatments, including intensive chemotherapy and allogeneic stem cell transplantation (allo-SCT) [2]. Systemic AML treatment has long been shaped by the prevailing belief that leukemic cells can only be eliminated by a “direct hit” against the malignant cell itself. In consequence of this dogma, cell-cycle active compounds such as cytosine arabinosides have been established as the backbone of most treatment protocols. Depending on the ability to tolerate such treatment, up to 80% of patients achieve a complete remission (CR) in response to these regimens [3] . However, without additional therapy virtually all patients relapse within a matter of months. Post-remission therapy in the form of additional chemotherapy or allo-SCT is therefore mandatory and frequently employed with the goal to eliminate residual leukemia cells that survive induction chemotherapy. Yet, many patients still relapse after post-remission therapy which highlights the need for novel strategies to more effectively combat AML.

Against the background of the “direct hit” dogma, harnessing the immune system to systemically attack AML cells has initially been considered to be of little benefit. This reckoning was fueled by the results of several AML vaccination studies which showed only a few significant clinical responses [4,5]. However, the success of allo-SCT foregrounded the importance of immunotherapeutic concepts in the management of this fatal disease. In recent years, an increasing number of immune system targeted agents have gained access to the clinical arena. With the advent of rituximab in the treatment of Non-Hodgkin lymphomas [6], passive immunotherapies targeting defined targets on tumor cells have become an essential component in the treatment of various hematologic malignancies. In addition, the dramatic impact of checkpoint inhibitors such as ipilimumab [7] and nivolumab [8] on the outcome of advanced melanomas have clearly shown that immunotherapy can result in durable cancer remissions, and that immunogenic cells represent promising, “tumor cell independent” therapeutic targets. Most recently, the bispecific T-cell engager blinatumomab was granted full approval by the Food and Drug Administration (FDA) to treat relapsed/refractory B-cell precursor acute lymphoblastic leukemia in adults and children after a phase 3 study showed a significant survival benefit for patients treated with blinatumomab compared to traditional chemotherapy [9]. This approval marks the first time the FDA has approved an immunotherapeutic agent for the treatment of acute leukemia since the approval of gemtuzumab ozogamicin, and rings in the beginning of a paradigm change in the management of this disease.

The goal of this review is to provide insight into novel immunotherapeutic principles that holds the promise of a paradigm shift in the management of AML.

## 2. Monoclonal Antibodies (mAbs)

### 2.1. CD33

CD33, a glycosylated transmembranous protein and member of the “sialic acid-binding Ig-related lectins” (siglecs, siglec-3), functions as an important mediator of cellular adhesion and interaction. High levels of CD33 expression have been reported on myeloid precursor cells in the bone marrow (BM) and on AML blasts, where expression of the CD33 antigen is found in up to 90% of cases [10]. CD33 therefore represents a promising target for AML therapy. Gemtuzumab ozogamicin (GO), a conjugate of a recombinant humanized CD33 antibody and the antitumor antibiotic calicheamicin, is one of various antibody-cytotoxic agent complexes that was initially designed to selectively target CD33 expressing leukemic cells. Due to its encouraging activity in single agent and combination clinical trials, GO was granted accelerated approval in 2001 but was then voluntarily withdrawn from the US market in 2010 after considerable toxicities, mainly consisting of substantial liver toxicity, were reported [11]. In 2011, the United Kingdom Medical Research Council published the results of a clinical trial (MRC AML 15) in which 1,113 de novo AML patients aged less than 60 years were randomized to receive induction chemotherapy with or without GO (3 mg/m^2^). Upon remission, 948 patients were randomized to receive consolidation chemotherapy alone or combined with GO. The investigators reported that the addition of GO to chemotherapy was safe but did not lead to any improvement in response or survival rates. However, predefined analysis by cytogenetics showed a significant survival benefit for AML patients with favorable, and a trend for benefit in patients with intermediate risk disease [12]. Another randomized trial conducted by the same group (MCR AML 16) randomized 1115 elderly patients with previously untreated AML or high risk myelodysplastic syndrome (MDS) to induction chemotherapy with or without GO (3 mg/m^2^). In this study, the addition of GO was not associated with any improvements in remission rate, 30- or 60-day mortality, or toxicity. However, the 3-year cumulative incidence of relapse and 3-year survival rate were both significantly improved in the GO arm [13]. Another trial investigated the benefits of low dose Ara-C (LDAC) compared to LDAC combined with GO (5 mg) in 495 elderly AML patients. The authors found that the addition of GO was associated with a significant improvement in CR rates (30% [LDAC+GO] vs. 17% [LDAC]) but that the 12-month overall survival (OS) was not improved [14]. Recently, a meta-analysis of individual patient data derived from five randomized trials, including 3325 patients with a median age of 58 years, demonstrated that the addition of GO to conventional induction chemotherapy is safe and associated with a significant survival benefit for patients without adverse cytogenetic risk factors [15]. Targeting CD33 on leukemic blasts with bispecific T-cell engagers (BiTEs) has shown promising activity in preclinical studies. BiTEs were specifically designed to bind to a surface target antigen on cancer cells and to CD3 on T-cells, thus bringing both cells to close proximity with the goal to induce T-cell activation and lysis of the attached cancer cell by cytotoxic granule fusion, cytokines release, and membrane perforation. AMG330, a CD3/CD33- bispecific T-cell engaging antibody showed promising pre-clinical activity against AML cells in vitro as well as in mouse models [16]. A phase 1 study of AMG330 in subjects with relapsed/refractory AML is currently ongoing (ClinicalTrials.gov Identifier: NCT02520427). Several other trials targeting CD33 are actively recruiting patients (Table 1).

### 2.2. CD123

Another promising target is represented by the Interleukin-3 receptor α chain (IL3Rα/CD123) which is overexpressed on leukemic stem cells and AML blasts compared to normal hematopoietic cells [17]. CSL362, a monoclonal antibody to CD123 demonstrated significant, antibody-dependent cell mediated cytotoxicity against AML blasts and was highly effective in reducing the leukemic burden in AML xenograft mouse models [18]. Results from a first in man, phase 1 study of CSL362 in patients with CD123 positive AML in complete remission at high risk for early relapse showed that CSL362 is safe, well tolerated, and durably depletes CD123 positive cells [19]. Phase 2 studies of CSL362 are currently under way (Table 1). SL-401, a novel CD123 targeted antibody, is currently being evaluated as a consolidation strategy in AML patients in CR1 or CR2 with high risk of relapse. Data from a multicenter, single-arm phase 2 trial showed that SL-401 is well tolerated and confers potent anti-leukemic activity with the potential to eliminate drug-resistant AML cells in patients with minimal residual disease (MRD) [20] (NCT02270463) (Table 1). XmAb14045 is a bispecific antibody that contains both a CD123 and a CD3 binding domain to activate T-cells for effective killing of CD123 expressing AML cells. A Phase 1 clinical trial of XmAb14045 for the treatment of AML and other hematologic malignancies is currently ongoing (NCT02730312) (Table 1). Similarly, flotetuzumab (also known as MGD006 or S80880) both recognizes CD123 and CD3 to redirect T-lymphocytes to eliminate CD123 expressing cells. Flotetuzumab effectively reduced leukemic burden in mouse AML xenograft models and was well tolerated in monkeys who were treated with continuous infusion of up to 1 µg/kg per day during a 4-week period [21]. In January 2017, flotetuzumab was granted orphan drug designation by the FDA. A phase 1 study of flotetuzumab in relapsed/refractory AML or intermediate-2/ high risk Myelodysplastic Syndrome (MDS) patients is under way (NCT02152956) (Table 1). JNJ-63709178, another CD3/CD123 targeted bispecific antibody was in early phase studies when a complete hold was instituted after several patients suffered serious adverse events.

### 2.3. CD133

CD133, also known as prominin-1, is a transmembrane glycoprotein that is primarily localized to the plasma membrane [22]. Expression of CD133 has been reported for a wide variety of tumor cells, including AML progenitors [23], and has been associated with resistance to chemotherapy and radiation [24]. Strategies to effectively target CD133 positive tumor cells are currently being explored in an effort to eliminate residual tumor cells that remain after conventional treatment. Recently, Rothfelder et al. reported that 293C3-SDIE, an FC-engineered CD133 monoclonal antibody, induced degranulation and lysis of primary CD133 positive AML cells by natural killer cells in allogeneic and autologous ex vivo settings. Interestingly, 293C3-SDIE exhibited no relevant toxicity against healthy BM cells. In xenotransplantation models, treatment with 293C3-SDIE led to the elimination of patient AML cells by NK cells from a matched human donor [25].

### 2.4. CD64

Another potential target for the delivery of cytotoxic agents is represented by CD64 (FcγRI), the high affinity receptor for IgG, which is expressed in several types of AML [26,27]. In vitro studies using the AML-related lymphoma cell line U937 showed that low nanomolar doses of Gb-H22/scFv (granzyme B fused to H22, a humanized single chain antibody fragment [scFv] specific to CD64) bound to CD64 positive U937 cells and induced apoptosis. Similar results were obtained in primary CD64 positive AML cells whereas CD64 negative AML cells were unaffected [28].

### 2.5. C-Type Lectin-Like Molecule 1

C-type lectin-like molecule 1 (CLL-1) is a transmembrane receptor that is expressed on the majority of myeloid blasts and stem cells derived from AML patients but not on normal tissues [29]. In a series of in vitro and in vivo studies, Zhao et al. showed that CLL-1 directed antibodies induced complement-dependent cytotoxicity against AML cell lines and primary cells, and furthermore reduced the tumor burden in AML xenograft mouse models [30].

### 2.6. Other Targets for Antibody-Directed Therapy

Recent evidence suggests that the CD98 glycoprotein plays a key role in mediating cellular interaction of AML cells with their microenvironment thus promoting leukemogenesis and leukemic cell maintenance. Using patient derived AML cells and mouse models, Bajaj et al. demonstrated that the CD98 directed antibody IGN523 blocks AML cell growth and reduces leukemic burden. Their findings strongly indicate that targeting CD98 could serve as a powerful tool to improve therapeutic targeting of AML cells [31]. Another promising target is represented by the fatty acid transporter CD36. Ye at al. showed that CD36 expressing leukemic progenitors are enriched in adipose tissue, such as in gonadal adipose tissue, where they are protected from the cytotoxic effects of chemotherapy suggesting a role for targeting CD36 as a novel strategy to hinder the development of treatment resistance [32,33]. Lines of evidence suggest that CD25 (Interleukin-2 receptor α) expression is increased in AML blasts and that its expression may be associated with an adverse outcome [34,35]. Further, methotrexate resistant subpopulations of the leukemic cell lines HL60 and MOLT4 exhibited elevated CD25 and TRAIL receptor 2 (TRAILR2)/Death receptor 5 (DR5) levels. Targeting CD25 as well as TRAILR2 or DR5 resulted in substantial cytotoxicity against leukemic cell lines and leukemic cells derived from AML, ALL, CML and CLL patient samples. CD25-based targeting may therefore represent and valuable strategy for targeting chemo-resistant leukemic cells [36]. CD38 (also known as cyclic ADP ribose hydroxylase) is a transmembrane glycoprotein that is expressed in most hematopoietic cells where it is involved in cell adhesion and signal transduction. When investigating CD38 expression in 304 AML patients, Keyhani et al. found that increased CD38 expression is associated with a favorable prognosis [37]. When investigating CD38 expression in AML cell lines, AML patient and healthy donor bone marrow samples, Dos Santos et al. reported a considerable variation among cell lines and AML patients whereas CD38 expression was more consistent in healthy bone marrow samples. The same group also investigated the effects of the CD38-targeted monoclonal antibody daratumumab against a range of AML cell lines and found that daratumumab-induced apoptosis was not correlated with CD38 expression levels. In AML patient xenografts, daratumumab significantly reduced leukemic burden in the peripheral blood and spleen, but not in the bone marrow. Intriguingly, the authors observed that daratumumab treated AML blasts displayed diminished CD38 surface expression indicating that the bone marrow microenvironment stalls the anti-leukemic effects of daratumumab [38]. Further studies to enhance the anti-leukemic activity of daratumumab might thus need to focus on disrupting the protective effects of the bone marrow microenvironment.

### 2.7. Targeting AML Stem Cells

Leukemia stem cells (LSCs), capable of giving rise to identical daughter cells and differentiated cells, perpetuate and maintain AML. It has become increasingly clear that LSCs are difficult to eliminate by the means of standard chemotherapy for which reason they represent the source of treatment resistance and relapse. The advent of immunotherapy in hematological malignancies has sparked hope for improved targeting of AML stem cells but many obstacles remain. For example, a clear characterization of the human AML stem cell phenotype is lacking. In recent years, several putative LSC targets such as the hedgehog and NFkB signaling pathways, MLL, CD44, CD47, CD33, CD96, CD123 and c-KIT have been proposed and tested in clinical trials [39]. To this end, encouraging experimental studies have recently been published for CD123. Han et al., for example, reported that SL-101, a CD123 targeted antibody conjugate, conferred remarkable anti-leukemic activity by sustained inhibition of protein synthesis, induction of apoptosis, blockade of IL3-induced STAT5 and AKT signaling, and inhibition of colony formation. In addition, the authors found that SL-101 significantly hindered repopulation of LSCs in patient derived xenograft models [40]. Similarly, dual targeting of CD19 and CD123 on leukemic blasts with chimeric antigen receptor T cells (CART) exhibited striking anti-leukemic activity in animal studies [41].

## 3. Increase Antigenicity of AML Cells by Hypomethylating Agents (HMAs)

The hypomethylating agents azacitidine (AZA) and decitabine (DEC) are the standard of care for management of patients with higher risk MDS [42] and are also frequently used off-label in AML patients deemed ineligible for intensive chemotherapy [43]. Despite clinical activity, only half of MDS patients respond to HMAs and the response is usually limited as most patient progress within 2 years of therapy with dismal survival [44]. In addition to their off-label upfront use in elderly and unfit patients with AML, HMAs are used in the setting of refractory or relapsed AML with a complete response (CR)/CR with incomplete count recovery (CRi) rate of approximately 16% [45]. Furthermore, HMA-based combinations with other forms of epigenetic therapy, particularly histone deacetylase inhibitors (HDACi), have been extensively studied in both AML and MDS but without clinical evidence of increased potency or synergism [46,47]. One important reason for suboptimal responses is that the underlying mechanism of action and resistance to HMAs are poorly understood [48].

**Figure 1:**
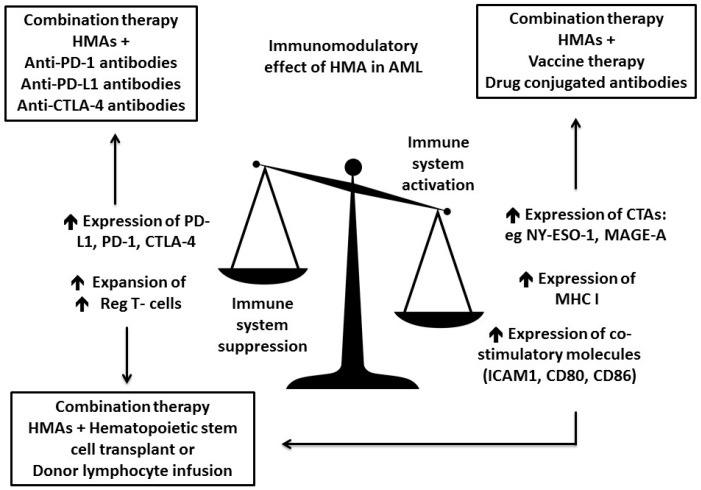
Hypomethylating agents (HMAs) and their role as immunomodulatory drugs in AML. HMAs possess both immuno-stimulatory, as well as immunosuppressive properties. They stimulate an immune response against AML blasts by increasing the expression of cancer testis antigens (e.g., NY-ESO-1 and MAGE-A), as well as important elements of the antigen-presenting machinery like the MHC I molecule and costimulatory molecules like ICAM1, as well as CD80 and CD86. On the other hand, HMAs can lead to immune escape of AML blasts through upregulation of immune checkpoints and their ligands, as well as regulatory T-cells. Combining the immunomodulatory effects of HMAs with other forms of immunotherapy holds the promise of a synergistic effect on the immune system. HMAs are currently combined with vaccines and drug-conjugated antibodies with the goal of increasing antigenicity and therefore AML blasts recognition and elimination by the immune system. Furthermore, combining HMA with checkpoint inhibitors might enhance the effect of checkpoint inhibitors in restoring immune surveillance. Lastly, combining HMAs with allo-HSCT or donor lymphocyte infusion is based on the hope that HMAs will enhance the graft versus leukemia effect (GVL) via enhanced antigenicity while limiting graft versus host disease (GVHD) by expansion of regulatory T-cells.

HMAs do not only lead to promoter hypomethylation of silenced tumor suppressor genes but were also found to have pleiotropic effects on cell differentiation, senescence, apoptosis, angiogenesis and the immune system [48]. Further, HMAs stimulate multiple aspects of the immune response against malignant cells by enhancing antigenicity (e.g., tumor antigen expression, processing and presentation) as well as T-cell priming and effector function [49]. HMAs were shown to limit hyper-activation of the immune system against malignant cells by up-regulating the PD-1/PD-L1 and CTLA-4/CD80/86 axis, as well as by expansion of T regulatory cell subsets (Figure 1) [49,50]. Their important role in modulating the immune response to AML holds promise for potential synergism with different forms of immune therapy and several HMA based combination are currently being evaluated in clinical trials (Table 1).

### 3.1. HMA Enhance Antigen Presentation

Cancer testis antigens (CTA) are expressed in adult testes and fetal ovaries but also in a variety of solid tumors where they trigger endogenous anti-tumor immune responses [51]. In contrast, AML blasts rarely express CTA as these genes are silenced by dense promoter hypermethylation. One exception, however, is the CTA preferentially expressed antigen in melanoma (PRAME), which is detected on AML blasts [52,53,54]. AZA, DEC and SGI-110 have been shown to induce the expression of multiple, previously suppressed CTAs (such as MAGE-A, NY-ESO-1 and SSX-2) per hypo-methylation of their promoter regions in AML cell lines and in AML xenograft models, as well as in primary AML blasts (Figure 1) [52,55,56,57,58]. HMA-induced expression of CTA in AML cells is sufficient for recognition of AML cells by CTA specific CD8+ cytotoxic T cells [55,57,58]. Furthermore, HMAs up-regulated the expression of important co-stimulatory proteins participating in antigen presentation, including the MHC class I molecule and the co-stimulatory molecules CD80 and CD86, as well as the intercellular adhesion molecule 1 (ICAM1) (Figure 1) [57,58,59]. However, HMAs alone are unlikely to trigger an adequate, endogenous immune response to eliminate leukemic cells. Therefore, several clinical trials are focusing on the combination between HMAs and vaccine therapy against CTA, drug conjugated antibodies directed towards tumor antigens, and adoptive cell therapy (Figure 1). Importantly, prior studies have not found an association of HMA-induced CTA expression and clinical responses to HMAs, suggesting that combining HMA with immunotherapy might be beneficial, including in patients who have previously failed HMA therapy [57]. A phase 1 study of an NY-ESO-1 peptide vaccine utilized protein CDX-1401, a fusion of a full length NY-ESO-1 protein sequence with a monoclonal antibody against DEC-205 (a surface marker present on many antigen presenting cells) combined with DEC in patients with AML and MDS (NCT01834248) [60]. Fusing NY-ESO-1 with an antibody against DEC-205 is supposed to enhance targeted delivery of NY-ESO-1 to the antigen processing machinery and subsequently enhance the efficacy of dendritic cell mediated T-cell priming. These NY-ESO-1 primed T cells will then more effectively target AML blasts expressing increased levels of NY-ESO-1 after being treated with DEC. For each cycle, patients were vaccinated 14 days prior and 14 days after receiving a 5-day course of DEC with the plan to administer 4 cycles. No significant side effects were observed and of a total of 9 patients included in this study, 5 patients had evidence of NY-ESO-1 specific CD4+ T-cell responses and 4 patients were found to have NY-ESO-1 specific CD8+ T-cell responses following vaccination. Another phase 1 trial examined the combination of the anti-CD33 targeted antibody drug conjugate SGN-CD33A with AZA or DEC as frontline therapy in 53 AML patients deemed unfit for intensive chemotherapy [61]. The response rate for the combination of SGN-CD33A and HMAs was 73% and of all responding patients, 47% achieved a minimal residual disease negative state. Based on these results, a phase 3 trial to test the combination between SGN-CD33A and HMAs (NCT02785900) is in progress (Table 1). Unfortunately, the FDA imposed a hold on clinical trials evaluating SGN-CD33A due to concerns of hepatotoxicity in patients who were treated with SGN-CD33A and received allo-SCT.

### 3.2. HMA Enhance Checkpoint Inhibition

Overexpression of the immune checkpoints PD-L1 on AML blast as well as PD-1 on BM stromal cells was found to lead to diminished antitumor T-cell responses and subsequent immune escape of AML blasts [50,62]. Furthermore, interferon-γ induced PD-L1 expression on AML cells is most prominent after initial treatment with chemotherapy possibly explaining high rates of relapse in AML despite initial remissions [63]. Importantly, HMAs also increased expression levels of PD-1, PD-L1 and CTLA-4 on peripheral blood (PB) mononuclear cells from MDS and AML patients as well as on leukemia cell lines (Figure 1) [50]. Patients resistant to HMA had relative higher increments in expression of the immune checkpoint genes compared to patients who responded to HMAs. This could not only explain the development of HMA resistance but also presented an opportunity for drug synergism when combining HMA with checkpoint inhibitors. Recently, early results of a phase 1 clinical trial combining AZA with the anti-PD1 antibody nivolumab in 51 AML patients, who had failed prior therapy, were presented at the American Society of Hematology 2016 Annual Meeting [64]. In this study, patients received AZA on days 1–7 and nivolumab on Day 1 and 14 with courses repeated indefinitely as tolerated. Median OS was 9.3 months, which compared favorably to historical survival with AZA-based salvage protocols in a similar patient population. Patients who responded had higher ratio of PD1 positive effector T-cells to PD1 positive regulatory T-cells in the BM microenvironment at baseline compared to non-responders. Several other clinical trials combining HMA with immune checkpoint inhibition are currently recruiting refractory and relapsed AML patients as well as elderly AML patients, who are unfit for intense chemotherapy (Figure 1). Some of these trials examine the combination of the anti-PD-1 antibodies nivolumab+AZA (NCT02397720), pembrolizumab+AZA (NCT02845297), as well as the combination of the anti- PD-L1 antibodies durvalumab+AZA (NCT02775903) and atezolizumab+ guadecitabine (NCT02892318) (Table 1).

### 3.3. HMA Might Enhance GVL Effect While Reducing GVHD

The goal of many allo-SCT trials is to enhance the graft versus leukemia effect (GVL) while limiting toxicities associated with graft versus host disease (GVHD). Sequential combination of allo-SCT followed by HMA (preemptively or at time of minimal/clinical relapse) could serve both goals by increasing the GVL through enhanced antigenicity of AML blasts as well as limiting GVHD by expanding regulatory T-cells, in addition to directly targeting leukemic cells by HMAs [65,66,67,68,69] (Figure 1). In a study of 27 patients with AML who had underwent reduced intensity allo-SCT, monthly courses of AZA were given and cytotoxic T-cell responses to candidate CTAs and circulating regulatory T-cells were measured [70]. HMA administration was found to not only increase the cytotoxic T-cell response to several CTA (including MAGE-A1 and WT-1) and expand the number for regulatory T-cells, but was also associated with low incidence of GVHD. A Phase 1/2 clinical trial of DEC followed by donor lymphocyte infusion in patients with AML, who relapsed after allo-SCT is currently recruiting participants (NCT01758367).

## 4. Modulation of the Leukemic Immune Microenvironment

Tumors generate a disabling immunosuppressive tumor microenvironment that limits the ability of the immune system to act against malignant cells. The immunosuppressive microenvironment begins forming as early as the first pre-malignant change. Thus, developing strategies to disrupt the immunosuppressive properties of the tumor microenvironment are necessary.

### 4.1. Small Molecule Immunomodulatory Drugs (IMiDs)

Small molecule immunomudulary drugs (IMiDs) are thalidomide analogues with immune-modulary, anti-angiogenic, anti-inflammatory and anti-proliferative properties. Lenalidomide, one of the best known thalidomide derivatives, is an orally bioavailable compound that has been FDA approved for the treatment of patients with multiple myeloma, MDS and mantle cell lymphoma. In vitro co-culture systems of endothelial cells and chronic lymphocytic leukemia cells showed that lenalidomide effectively altered the leukemic microenvironment and inhibited CLL cell survival by disruption of the cross-talk between leukemic and endothelial cells [71]. Recent studies suggest that lenalidomide has clinical activity in AML although the patient population that would benefit the most from lenalidomide needs to be better defined [72,73,74]. Several studies of lenalidomide in AML are currently ongoing, including the evaluation of lenalidomide as a consolidation and maintenance strategy in elderly AML patients following standard induction (NCT01578954).

### 4.2. Immunosuppressive Factors Expressed and Secreted by the Tumor or Tumor Microenvironment

Most of soluble immunosuppressive factors are secreted by the cells in the tumor microenvironment (stromal cells or infiltrating myeloid cells as described below) in response to the tumor or the tumor itself. In addition to cell–cell interactions that can inhibit effector lymphocytes, soluble factors can suppress the local immune response, which creates a hostile environment for infiltrating effector cells within the tumor. Indoleamine 2,3-dioxygenase (IDO), transforming growth factor-β (TGFβ), interleukin-10 (IL-10), vascular endothelial growth factor (VEGF), galectins, and IL-33 have been the most studied so far. IDO is the rate-limiting enzyme for the catabolism of the essential amino acid tryptophan. High levels of IDO reduce tryptophan levels and generate tryptophan metabolites which suppress T cell activity [75]. Phase 1 and 2 trials with IDO inhibitors are currently being carried out in melanoma, MDS as well as in AML patients (Table 1). Increased local and systemic TGFβ levels are associated with progression and poor clinical outcome in several tumors which make it an attractive therapeutic target. A small molecule kinase inhibitor blocking TGFβ receptor (LY2157299, Lilly^®^) is in clinical development in melanoma. The hypoxic environment of tumor also induces other modulators such as IL-10, IL-27, IL-33 and VEGF [76]. IL-33 can be detected not only in the tumor environment, but also in the serum of cancer patients. IL-33 levels are increased in the serum of lung and gastric cancer patients and correlate with disease stage, and expression levels of IL-33 and ST2 correlate with tumor grade and inferior survival of glioblastoma patients, suggesting that IL-33 may be a negative prognostic marker for these types of cancer [77,78,79]. As for hematological diseases, the IL-33/ST2 signaling pathway has been shown to increase the development of both BCR-ABL1-positive and -negative MPNs. Increased levels of nuclear IL-33 protein are present in biopsies of BCR-ABL1-negative MPN patients, and high amounts of circulating soluble ST2 levels were detected in the plasma from CML patients, compared to controls [80,81,82]. While cytokine blockade is currently applied for the treatment of inflammatory disorders, it remains to be investigated whether a blockade of the IL-33/ST2 pathway may represent a valid approach for the therapy of established IL-33-dependent tumors.

### 4.3. Myeloid-Derived Suppressor Cells

Myeloid-derived Suppressor Cells (MDSC) are a heterogeneous group of immature myeloid cells with potent immune suppressing activity [83]. MDSCs are characterized by the expression of the myeloid markers CD11b and CD33, and absence of HLA-DR8. There are two subsets of MDSCs: the monocytic MDSCs that are CD15 negative and the granulocytic MDSCs that are CD15 positive [83]. Presence of high numbers and frequencies of MDSCs in the tumor microenvironment has been associated with tumor progression [84], worse outcomes [85] and poor response to new immunotherapeutic approaches [86]. MDSCs directly suppress effector CD8+ T cells via T cell receptor (TCR) downregulation, expression of immunomodulatory enzymes such as Arginase-1, iNOS and the production of ROS [83,87]. Although MDSCs have mostly been studied in solid tumors growing evidence suggests that MDSCs are increased in patients with MDS [88]. Further, Pyzer et al. demonstrated that MDSCs are elevated in the PB of AML patients. Using tracking studies, the authors also showed that tumor-derived extracellular vesicles (EVs) are taken up by myeloid progenitors which leads to the selective proliferation of MDSCs. This process is largely driven by the Mucin 1 (MUC1) onco-protein which induces the expression of c-myc in AML cells and EVs [89]. Strategies to suppress MDSCs in vivo represent a promising approach to improve the virtue of immune-based therapies in AML [90].

### 4.4. Tumor Associated Macrophages

Tumor-associated macrophages (TAMs) are abundant in most human and murine cancers and are pro-tumorigenic [91]. TAMs express high levels of IL-10 and low levels of IL-12 with expression of the mannose receptor and scavenger receptor class A (SR-A) [92]. There is clinical evidence that an abundance of TAMs in the tumor microenvironment is correlated with poor prognosis [93]. It has recently been shown that AML cells polarize macrophages towards a leukemia supporting state in a Growth factor independence-1 (GFI1) dependent manner [94]. Targeting TAM with Anti-CSF-1R Antibody has been proposed in experimental models of colon carcinoma with promising results [95].

### 4.5. Tumor Associated Neutrophils

There is evidence that tumor associated neutrophils (TANs) promote primary tumor growth in mouse cancer models by enhancing angiogenesis and increasing immune suppression [96,97,98]. The role of TANs in the malignant BM niche has yet to be defined.

### 4.6. Regulatory T Cells

Regulatory T cells (Tregs) play a crucial role in maintaining peripheral tolerance and preventing autoimmunity. However, they also represent a major barrier to effective antitumor immunity and immunotherapy. Consequently, there has been considerable interest in developing approaches that can selectively or preferentially target Tregs in tumors, while not impacting their capacity to maintain peripheral immune homeostasis. Suppressive cytokines, such as IL-10, IL-35, and TGFb, are secreted by Tregs and required for their maximal suppressive function [99]. IL-35 contributes to the optimal suppressive activity of Tregs. In an experimental melanoma model, tumor growth was reduced in mice treated with an IL-35-neutralizing mAb or in mice harboring a Treg-restricted deletion of Ebi3 that prevents IL-35 production [100]. Treg-mediated cytolysis of NKs and CD8+ T cells via granzyme B and perforin may also be a relevant mechanism within tumors [101]. Lines of evidence suggest that the frequency of BM and PB Tregs are greater in AML patients compared to healthy controls. In mice, Tregs accumulate in leukemic sites and impede the proliferative and cytolytic capacity of adoptively transferred anti-AML reactive CTLs [102]. Depletion of CD25 (IL-2 receptor α-chain)–expressing Tregs by the administration of IL-2 diphtheria toxin results in temporary tumor regression associated with increased CTLs at tumor sites. Combination therapy with IL-2 diphtheria toxin and anti-AML adoptive CTL transfer not only reduces tumor mass but also improves survival in mice. Moreover, mice display resistance to AML cells on re-challenge, implying the development of effective adaptive immunity [102,103]. Clinical studies with anti-CD25 monoclonal antibodies in patients with several cancers have led to no or only transient reductions in circulating Tregs following its administration, which has tempered enthusiasm for this strategy [104]. In AML, clinical studies are currently underway to investigate whether T cell responses to tumor vaccines are augmented in AML patients following depletion of Tregs [104].

### 4.7. Tumor Expressing Inhibitory Molecules, Cytotoxic CD8+ T Cells Exhaustion and Checkpoint Inhibitors

Therapeutic blockade of immune checkpoint pathways, in particular cytotoxic T-lymphocyte associated protein 4 (CTLA-4) and programmed-death 1 (PD-1), has become a paradigm-shifting treatment in solid tumor oncology. This therapeutic approach evolved from the recognition that tumors can evade the host immune system by high-jacking immune checkpoint pathways, such as CTLA-4 and PD-1 pathways [105]. CTLA-4, PD-L1 and PD-L2 are expressed by either stromal or immune cells in the microenvironment of tumor cells [106]. The engagement of checkpoint receptors on the surface of CD8+ T cells by their cognate ligands [B7-1 and B7-2 for CTLA-4, PD ligand 1 (PD-L1) and PD-L2 for PD-1] leads to the temporary downregulation of CD8+ T cell function, and to their exhaustion [106,107]. Of note, inhibition of T-cell activity by PD-1/PD-L1 engagement appears to be stronger than by CTLA-4 engagement [108]. Hematologic malignancies, many of which are known to have clinically exploitable immune sensitivity, are a natural target for this type of treatment [109]. Several clinical trials of checkpoint blockade have been carried out in hematologic malignancies, with preliminary results strongly suggesting therapeutic usefulness of this approach across several tumor types. In particular, the results of PD-1 blockade in Hodgkin lymphoma have been remarkable [110,111]. In non-Hodgkin lymphomas, response rates of 36% in diffuse large B-cell lymphoma (DLBCL) and 40% in follicular lymphoma (FL) were achieved with nivolumab, however, it is to be noted that these results were achieved in combination with Rituximab [112]. In multiple myeloma (MM), there has been no objective response in an initial phase 1 trial for checkpoint blockade as single agent. A phase 2 study of the humanized anti–PD-1 mAb pembrolizumab (MK-3475) with lenalidomide post-autologous HCT (NCT02331368) and a phase 1/2 study of pembrolizumab plus pomalidomide/dexamethasone in refractory MM (NCT02289222) are ongoing. In addition, the anti–PD-1 mAb pidilizumab (CT-011) is currently being evaluated in combination with vaccination post- autologous HCT (NCT01067287), as well as with lenalidomide in refractory MM patients (NCT02077959). Activity in myeloid malignancies has only recently been evaluated knowing that PD-L1 is expressed on MDS blasts, possibly at a higher level in high-risk and in more refractory disease. Furthermore, it has been shown that PD-L1 expression is enhanced by treatment with hypomethylating agents [50,113]. In consequence, a phase 1 trial to evaluate PD-L1 blockade in subjects with MDS is currently accruing. Although there are no dedicated AML trials yet, the anti-CTL-4 antibody ipilimumab has been evaluated in patients with persistent or progressive hematologic malignancies, including AML, after allo-SCT [114]. In this study, among 22 patients who received a dose of 10 mg ipilimumab per kilogram, 5 (23%) had a complete response, 2 (9%) had a partial response, and 6 (27%) had decreased tumor burden. The best responses were seen in patients with leukemia cutis and Hodgkin lymphoma. Most patients showed immunological responses as well. However, numerous questions remain and toxicities were not negligible. For example, the authors observed immune-related adverse events in 6 patients (21%), including one death. GVHD that precluded further administration of ipilimumab was observed in 4 patients (14%). Comprehensive reviews of all immune-related adverse events observed with immune checkpoint blockade has recently been published [8,115]. Toxicities encompass severe auto-immune reactions that can affect all organs/tissues. The most frequent organs affected are skin, gastrointestinal tract, pulmonary, and joints, while the most severe immune-related adverse events involve the gastrointestinal tract and endocrine glands. Toxicities are less severe with PD-1 blockade as compared to CTLA-4 blockade and less with PD-L1 blockade as compared to PD-1 blockade. Of note, hematological syndromes are possible in patients with solid tumors but appears to occur more frequently in patients with lymphoma and include red cell aplasia, autoimmune neutropenia or pancytopenia.

## 5. Conclusions

Recent advances in our understanding of the complex interactions between the immune system and cancer cells has opened the door to novel treatment strategies for AML. These concepts have mostly emerged from the knowledge that the immune system can inhibit, shape and/or support cancer in a multistep fashion (commonly referred to as “cancer immuno-editing”) [116], and the realization that standard chemotherapeutic agents can exhaust immunosuppressive cells, including regulatory Tregs and myeloid-derived suppressive cells, thereby augmenting antitumor immune responses. To this end, multi-parameter flow cytometry on AML bone marrow specimens demonstrated that total T-cell and T-cell subpopulations are largely preserved in newly diagnosed and relapsed AML patients and therefore subject to therapeutic manipulation via checkpoint receptors such as PD-1 and OX40 [117]. Against this background, combining immunotherapy with conventional AML treatments holds the promise to more effectively eradicate leukemic cells compared to standard therapy alone. Several studies of immune-based therapies in AML and related diseases showed encouraging clinical activity in the setting of acceptable side effects and illustrated that these approaches have the potential to significantly enhance the current armamentarium for AML treatment. Data that was recently presented at ASH showed that inhibition of PD-1 with nivolumab in combination with azacitidine is safe and clinically beneficial in previously untreated patients with high risk MDS [118], a disease closely related to AML. Despite encouraging progress, major challenges on the way to establish immunotherapy as an integral element of AML therapy remain and are, at least in part, rooted in patient specific disparities in age, comorbidity and functional status, paired with the enormous molecular heterogeneity and complex clonal architecture of AML. For example, several immunotherapy trials have reported heterogenous and unconventional response patterns with regard to tumor regression and response times. Specifically, when compared to standard cytotoxic therapies which directly target cancer cells and therefore frequently lead to rapid reductions in tumor burden, responses to immunotherapy may take several months and frequently occur after an initial episode of “stable disease” or a temporary increase in total tumor burden (“pseudo-progression”) [119]. Therefore, accounting for the unique modes of action of immunotherapeutic agents, novel response evaluation criteria are needed to (i) accurately capture and report immunotherapeutic responses in a standardized fashion and (ii) avoid premature/inappropriate discontinuation of therapy. Additional challenges are represented by the identification and management of immune-related side effects. Although these events can occur in any organ, gastrointestinal, hepatic and dermatologic side effects, as well as endocrinopathies, are most commonly reported. Standard approaches to identify and treat immune-related adverse events, however, are lacking and any inflammatory process is therefore almost uniformly managed by the early initiation of corticosteroids. Additional hurdles are represented by staggering treatment costs. Immunotherapies belong to the most expensive group of cancer therapeutics and it remains unclear how patient- and disease-specific characteristics should guide the choice of immunotherapy. In addition, questions remain regarding their implementation in the curative, adjuvant and maintenance setting. In order to establish a role for immune-based therapies in the management of a highly heterogenous disease as AML, costly and large, randomized trials are needed which requires the identification of adequate biomarkers to help predict treatment response and toxicities, and to accurately select patients for accrual.

## Figures and Tables

**Table 1:** Immune-based therapeutic concepts under active development for the treatment of AML.

Target	Drug	Trial Phase	Patient Population	Single Agent/Combination	Ref./Identifier	Status
CD33	IMGN779	I	Adult patients with relapsed/refractory CD33+ AML	Single agent	NCT02674763	Recruiting
CD33	Gemtuzumab ozogamicin	II	Patients up to 70 years with AML induction/re-induction failure, AML in CR1 with poor cytogenetics, AML in 2nd CR with MRD, AML in 3rd CR, AML in refractory relapse but ≤25% BM blasts, MDS with >6% BM blasts at diagnosis, secondary MDS with ≤5% BM blasts at diagnosis Note: disease must express >/=10% CD33+ for patients with AML	Combination with busulfan and cyclophosphamide	NCT02221310	Recruiting
CD33	AMV564	I	Adult patients with relapsed/refractory AML	Single agent	NCT03144245	Recruiting
CD33	SGN-CD33A	III	Adult patients with newly diagnosed, previously untreated intermediate or adverse risk de novo or secondary AML	Combination with azacitidine or decitabine	NCT02785900	Recruiting
CD123	SGN-CD123A	I	Adult patients up to 74 years with relapsed/refractory CD123-detectable AML following at least 2 but no more than 3 prior regimens; patients may be eligible after only 1 previous regimen if in a high risk category	Single agent	NCT02848248	Recruiting
CD123	XmAb14045	I	Adult patients with primary or secondary AML , B-cell Acute lymphocytic leukemia (ALL), blastic plasmacytoid dendritic cell neoplasm (BPDCN), Chronic myeloid leukemia (CML) in blast phase, resistant or intolerant to tyrosine kinase inhibitors; patients with relapsed or refractory disease with no available standard therapy	Single agent	NCT02730312	Recruiting
CD123	JNJ-56022473 (CSL362)	II/III	Elderly patients, 65 years or older with de novo or secondary AML	Combination with decitabine	NCT02472145	Recruiting
CD123	MGD006	I	Adult patients with primary or secondary AML or MDS with an International prognostic scoring system (IPSS) category of intermediate 2 or high risk	Single agent	NCT02152956	Recruiting
CD123	SL-401	I/II	Adult patients with AML in first or second CR or CRi	Single agent	NCT02270463	Recruiting
PD-L1	Durvalumab (MEDI4736)	II	Adult patients with MDS or elderly patients (≥65 years) with newly diagnosed de novo AML or secondary AML	Combination with azacitidine	NCT02775903	Recruiting
PD-L1	Atezolizumab	I	Adult patients with relapsed refractory AML; elderly patients with treatment naiive AML who are unfit for induction chemotherapy	Combination with guadecitabine	NCT02892318	Recruiting
PD-1	Nivolumab	II	Adult patients with relapsed/ refractory AML	Combination with azacitidine; Combination with ipilimumab and azacitidine	NCT02397720	Recruiting
PD-1	Pembrolizumab	II	Adult patients with relapsed/ refractory AML	Combination with azacitidine	NCT02845297	Recruiting
CTLA-4	Ipilimumab	I	Adult patients with relapsed/refractory AML or MDS; Elderly patients (≥75 years) with treatment naïve de novo or secondary AML	Combination with decitabine	NCT02890329	Recruiting
IDO	Indoximod	I/II	Adult patients with newly diagnosed AML	Combination with “7+3”	NCT02835729	Recruiting
